# Comparing the effect of different sample conditions and spectral libraries on the prediction accuracy of soil properties from near- and mid-infrared spectra at the field-scale

**DOI:** 10.1016/j.still.2021.105196

**Published:** 2022-01

**Authors:** T.S. Breure, J.M. Prout, S.M. Haefele, A.E. Milne, J.A. Hannam, S. Moreno-Rojas, R. Corstanje

**Affiliations:** a Rothamsted Research, Harpenden AL5 2JQ, United Kingdom; b Cranfield University, Cranfield, Bedfordshire MK43 0AL, United Kingdom; c Gs Growers Ltd, Ely CB7 5TZ, United Kingdom

**Keywords:** In-situ spectroscopy, Local-regional scale, National Soil Inventory, Partial least-squares regression, Spiking

## Abstract

The prediction accuracy of soil properties by proximal soil sensing has made their application more practical. However, in order to gain sufficient accuracy, samples are typically air-dried and milled before spectral measurements are made. Calibration of the spectra is usually achieved by making wet chemistry measurements on a subset of the field samples and local regression models fitted to aid subsequent prediction. Both sample handling and wet chemistry can be labour and resource intensive. This study aims to quantify the uncertainty associated with soil property estimates from different methods to reduce effort of field-scale calibrations of soil spectra. We consider two approaches to reduce these expenses for predictions made from visible-near-infrared ((V)NIR), mid-infrared (MIR) spectra and their combination. First, we considered reducing the level of processing of the samples by comparing the effect of different sample conditions (in-situ, unprocessed, air-dried and milled). Second, we explored the use of existing spectral libraries to inform calibrations (based on milled samples from the UK National Soil Inventory) with and without ‘spiking’ the spectral libraries with a small subset of samples from the study fields. Prediction accuracy of soil organic carbon, pH, clay, available P and K for each of these approaches was evaluated on samples from agricultural fields in the UK. Available P and K could only be moderately predicted with the field-scale dataset where samples were milled. Therefore this study found no evidence to suggest that there is scope to reduce costs associated with sample processing or field-scale calibration for available P and K. However, the results showed that there is potential to reduce time and cost implications of using (V)NIR and MIR spectra to predict soil organic carbon, clay and pH. Compared to field-scale calibrations from milled samples, we found that reduced sample processing lowered the ratio of performance to inter-quartile range (RPIQ) between 0% and 76%. The use of spectral libraries reduced the RPIQ of predictions relative to field-scale calibrations from milled samples between 54% and 82% and the RPIQ was reduced between 29% and 70% for predictions when spectral libraries were spiked. The increase in uncertainty was specific to the combination of soil property and sensor analysed. We conclude that there is always a trade-off between prediction accuracy and the costs associated with soil sampling, sample processing and wet chemical analysis. Therefore the relative merits of each approach will depend on the specific case in question.

## Highlights

• Two methods that minimise calibration expenses for predictions from soil infrared spectra at the field-scale were contrasted.
• Results showed the potential to reduce time and cost implications for predictions of soil organic carbon, clay and pH.
• Uncertainty in organic carbon, pH and clay predictions increased by 0–76% under reduced sample processing.
• Uncertainty increased by 54–82% for spectral libraries and by 29–70% when spectral libraries were spiked.
• Available P and K could only be predicted with moderate accuracy from milled samples at the field-scale.

## Graphical Abstract

**Figure:**
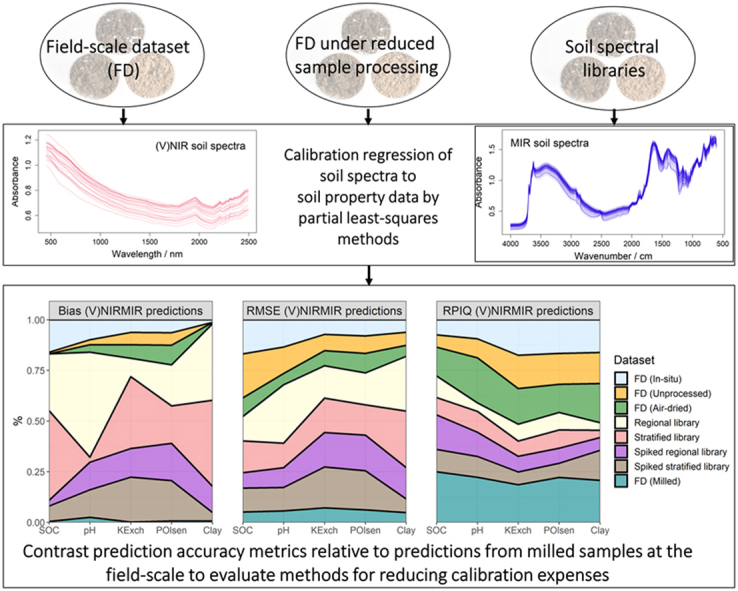

## 1 Introduction

Farmers are interested in the spatial variation of soil properties because this helps them explain the variation in crop performance and so infer appropriate interventions. Mapping subfield soil variation in the traditional manner (i.e. analysed by wet chemistry analysis) is usually deemed too expensive to obtain the accuracy required for precision agriculture (Muhammed et al., 2017). Improvements in technology, mean proximal and remote soil sensing (for example, using visible (V), near-infrared (NIR) and mid-infrared (MIR) spectroscopy) now offer an alternative and less resource demanding approach to predict soil variation than measurements based on wet chemistry (Viscarra Rossel and Bouma, 2016). Due to the reduced labour and monetary inputs, soil spectroscopy can be implemented at finer sampling scales than traditional sampling. For example, at a 10 m scale, which is reported to be necessary to characterise spatial and temporal soil variability for site-specific management (McBratney et al., 1996). Despite the practical advantages of using soil spectral measurements over wet chemistry, issues of efficiency still need to be addressed before wide-scale adoption is practical (Reeves, 2010). These largely relate to reducing sample processing and using spectral libraries to minimise resource input under the constraint that to be practically useful, however, they should maintain accuracy near to that of laboratory methods (Viscarra Rossel and McBratney, 1998).

The common methodology of soil preparation before the measurement of soil reflectance spectra includes air-drying and sieving (< 2 mm) and for MIR milling (< 100 *μ*m) of the soil samples. Minimizing the sample processing can reduce the accuracy in the soil property predictions due to effects of particle-size, aggregation and water content on spectroscopy measurements. A number of studies have researched these effects for (V)NIR/MIR soil spectroscopy. For example, studies analysed the effect of different particle sizes (Nduwamungu et al., 2009, LeGuillou et al., 2015, Coutinho et al., 2019, Wijewardane et al., 2020), soil water content (Bogrekci and Lee, 2006, Minasny et al., 2011, Ji et al., 2015) and in-field (V)NIR measurements (Stevens et al., 2008; Gras et al., 2014) on soil spectroscopy predictions.

Within the soil IR spectroscopy discipline, there have been efforts to develop spectral libraries (a point-dataset with paired reflectance and wet chemistry measurements) at local, regional, continental ( Shepherd and Walsh, 2002; Stevens et al. 2013; ViscarraRossel and Webster, 2012; Stevens and Ramirez-Lopez, 2020) and even global scales. Where traditional soil survey data already exists, creating a spectral library has the potential to minimise the effort of developing field-scale calibrations.

Ideally, one would consult existing literature to infer a quantified effect on prediction accuracy of using either reduced sample processing or spectral libraries to minimize calibration expenses. However, comparison across literature is hampered by differences in case-study characteristics (e.g. overall variance of soil properties and their counterparts in the calibration and validation set) and methods (e.g. number of samples with wet chemistry used in the calibration, chemometric models considered, (cross-)validation techniques used etc.). For example, due to increased availability of portable MIR spectrometers, recent studies have explored the accuracy of in-situ MIR measurements. However, the comparison between these studies is not straightforward as there are differences in the number of replicate measurements taken (Webster et al., 2016, Hutengs et al., 2019), whether or not in-situ means < 2 mm sieved soils (Webster et al., 2016), the MIR spectrometer manufacturer may be different (Dhawale et al., 2015, Ji et al., 2016, Webster et al., 2016) and the range of wave numbers can vary (Dhawale et al., 2015, Ji et al., 2016).

Prediction accuracy from spectral libraries at a local-scale have also been shown to be affected by different instruments or laboratory conditions, under-representation of the local soil type and differences in lithology, climate and other soil forming factors (Wetterlind and Stenberg, 2010, Ge et al., 2011, Guerrero et al., 2014). To overcome some of the limitations of using spectral libraries, Brown (2007) developed an approach to compute adequate models for new local target sites by adding local samples to a spectral library, which has been described as ‘spiking’ by (Viscarra Rossel et al., 2009). Spiking has been shown to improve prediction accuracy (Wetterlind and Stenberg, 2010; Guerrero et al., 2010, Guerrero et al., 2014, Guerrero et al., 2016; Seidel et al., 2019).

We were unaware of any research that examined the effects of in-situ sensing, particle size variation, aggregation and soil water content on spectral measurements for both (V)NIR and MIR spectroscopic predictions within a single study. Furthermore, contrasting the effect on prediction accuracy of reduced sample processing with that of spectral libraries on a single dataset will advance our understanding of when one approach could be preferable over the other. To that end, we explored the following questions:

• What is the difference in accuracy between soil property predictions from (V)NIR, MIR and (V)NIRMIR spectroscopy measurements taken on in-situ, unprocessed (i.e. fresh), air-dried or milled soil samples?
• If we were to use a spectral library rather than a field-scale calibration, does subset selection from a national spectral library by region or pedological characteristics minimise the prediction error?
• What can we learn from these findings to determine the best way in which to reduce laboratory, sampling preparation and handling efforts whilst minimising the loss in prediction accuracy?

## 2 Methods

**Fig. 1:**
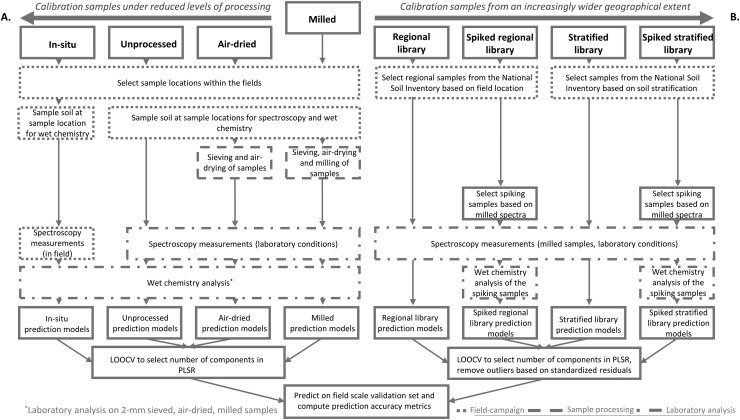
A schematic showing two strategies to reduce the effort required to make predictions about soil properties from visible-near-infrared ((V)NIR) and mid-infrared (MIR) reflectance spectra. The first is to reduce the processing of the soil samples used for calibration and those used for prediction (in-situ, unprocessed and air-dried). The second uses soil spectra from a National Soil Inventory (NSI). In this case the soils for prediction must be air-dried, sieved and milled to accord with those from the NSI. They can be chosen according to how representative they are, in this study based on geographic location (Regional library) or soil type (Stratified library). In both cases we also consider ‘spiking’ the library set with soils from the field-scale dataset for which we wish to predict soil properties. PLSR stands for partial-least squares regression, the method of regression used in this study. LOOCV stands for leave-one-out cross-validation, the procedure used in this study to select the final model for prediction.

Using soil samples from four fields within the Cambridgeshire fens in the UK, we evaluated two approaches for reducing the expense associated with soil variable predictions made from visible-near-infrared ((V)NIR), mid-infrared (MIR) spectra and their combination. The first considers reducing effort related to sample processing (Fig. 1A) and the second by using regional and stratified soil spectral libraries (with and without spiking) (Fig. 1B). The sample processing steps ranged from standard laboratory processing of soil for spectral analyses (air-dried, sieved and milled) to a gradual reduction of the laboratory processing effort (removing milling, sieving and air-drying) to taking spectral measurements in the field without sample processing. Wet chemistry was conducted on the field-scale dataset, which was then split into calibration (75%) and validation (25%) subsets. For the reduced sample processing analysis, the calibration samples were used to develop regression models between the measured soil properties and the spectra for each processing method.

We used two methods to subset a national soil inventory (NSI) into spectral libraries. Samples were selected i.) in close geographical extent to the field-scale dataset (the regional library) and ii.) by the two soil types found at the field site (the stratified library). Representative samples from the field-scale calibration subset were selected to spike the regional and stratified libraries. Regression models were developed for the regional and stratified libraries (and spiked versions).

All prediction models were applied to the field-scale validation subset and model accuracies computed. Details of data collection, processing and analysis of the various datasets are presented below.

### 2.1 Formation of spectral libraries

#### 2.1.1 Field-scale dataset

The four experimental fields used in this study make up the field-scale dataset and are located within the Cambridgeshire fens, south-east of England (UK). The fens contain complex soils which are a combination of peat with underlying alluvial and marine silts that became elevated features in the landscape due to peat oxidation and shrinkage (Hodge et al., 1984). The two soil types present are classified according to the World Reference Base taxonomy as a drainic sapric Histosol (dr sa HS) and a mollic Gleysol (mo GL) (IUSSWorkingGroup, 2015). Field 1, covering 8.2 ha, with British National Grid reference: TL607880, lies adjacent to Field 2 which covers 16.9 ha. Field 3 lies 8.3 km south-west, covering 5.1 ha and Field 4 lies 7.5 km south of Field 1 and Field 2, covering 8.9 ha. Three soil cores of topsoil (0–25 cm) were taken within a 0.5 m × 0.5 m quadrat at 25 sampling locations in each field. The fields were sown with lettuce which do not have a substantial root system, and any previous thatch layer was mixed in by tillage. For each sampling location, the three soil cores were bulked and mixed for laboratory analysis and spectral study, described in detail below (see also (Breure et al., 2021)). Direct spectral measurements of the soil surface were also taken at the sample locations in each field. Given the restricted number of samples for each field, we considered them as a single dataset. Since three locations had incomplete measurements we continued the analysis with a field-scale dataset where n = 97.

#### 2.1.2 Spectral library subsetting

We formed two spectral libraries. The samples that make up the two spectral libraries are a subset of the National Soil Inventory (NSI) dataset of England and Wales (McGrath and Loveland, 1992). The topsoil samples (0–15 cm) were taken as part of a 5 km × 5 km grid-based soil survey from 1979 to 1983. A full description of the survey methods, analytical methods and available data is given in the LandIS database (www.landis.org.uk; (Proctor et al., 1998)).

**Fig. 2:**
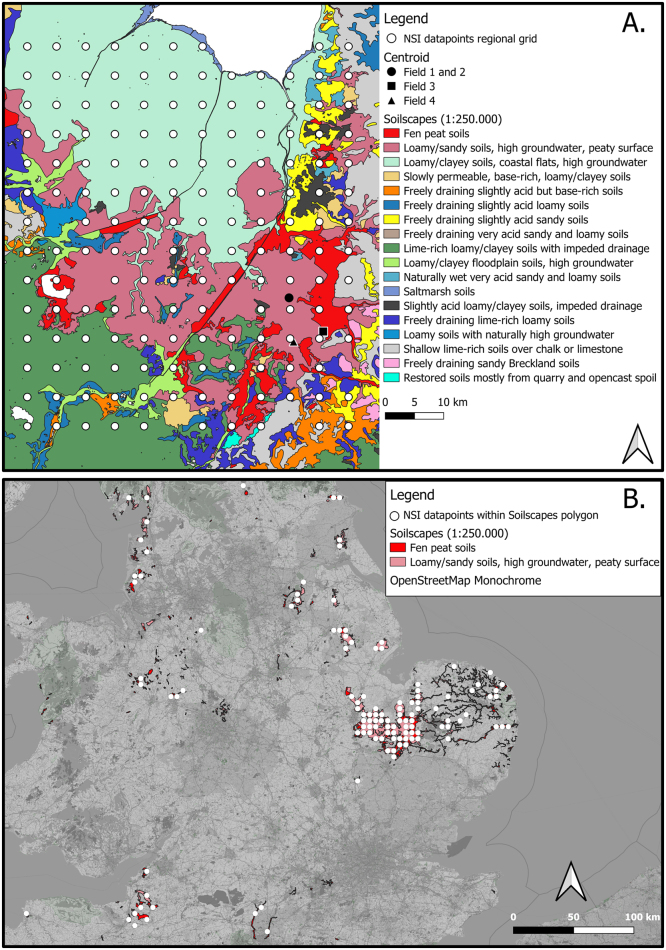
A.) National Soil Inventory (NSI) samples selected with the regional approach overlaid on the SoilScapes (1:250.000) dataset with the centroids of the study fields, B.) NSI samples selected with the stratification approach, overlaid on the SoilScapes polygons of the two major soil types that occur in the study fields.

The two spectral libraries were selected according to two different methodologies, and we refer to these as the regional library and stratified library (Fig. 2):

#### 2.1.2 1. Regional library

Based on the SoilScapes dataset (1:250,000 scale) (Farewell et al., 2011) a regional grid was selected around the case study area. We classified our field-scale dataset by two soil type descriptions: ‘Fen peat soils’ and ‘Loamy and sandy soils with high groundwater and a peaty surface’. We placed the regional grid such that it was centred around these two soil types within our case-study area and encompassed the fields sampled (Fig. 2A). The grid size was 65 by 55 km. The furthest distance from a field to a grid node was 68 km and the closest distance 4.5 km. The total number of samples was 159.

#### 2.1.2.2 Stratified library

The NSI dataset was stratified by the two dominant soil types found within the case-study area using the SoilScapes dataset. We extracted all of the NSI samples that lay within either a ‘Fen peat soil’ or ‘Loamy and sandy soils with high groundwater and a peaty surface’ polygon (Fig. 2B). The total number of samples was 109.

### 2.2 Sample air-drying, sieving and milling

The soil samples for the field-scale dataset were air-dried for 7 days, aggregates were crushed in a roller mill and passed through a < 2 mm sieve. The samples were then placed in a stainless steel cup together with a disk and milled for 35 s at 960 rotations per minute in a TEMA Machinery Ltd mill to a powder (< 100 *μ*m) (Northants, UK).

The soil samples of the NSI, which we used to make spectroscopy measurements, were available stored as a powder (< 100 *μ*m) in plastic sample bottles in the Rothamsted archive.

### 2.3 Wet chemistry analysis

The following laboratory methods were used for the samples from the field-scale dataset: Total carbon (C) (%) was determined by combustion (Dumas method) using an elemental analyser by LECO (TruMac Combustion Analyser, Michigan USA). Total C was assumed to represent total organic C as these soil types are unlikely to contain substantial amounts of carbonates. Available potassium (K) (mg kg^−1^) was determined by ammonium nitrate extraction and Inductively Coupled Plasma–Optical Emission Spectrometer (ICP–OES) (MAFF, 1986). Available phosphorus (P) (mg kg^−1^) was measured by the standard Olsen method (Olsen et al., 1954). The pH was measured in a 1:2.5 ratio of H_2_O. Particle-size fractions (%) were determined by laser diffraction (Breure et al., 2021).

For the NSI dataset, the laboratory methods for the soil properties of interest were measured as follows: Organic carbon (%) by loss-on-ignition for soils that were estimated to contain more than 20% organic carbon (Avery and Bascomb, 1982), otherwise by dichromate digestion (Kalembasa and Jenkinson, 1973). Extraction methods for extractable potassium (K) and phosphorus (P) were standardized by their volume rather than their weight (MAFF, 1988). Extractable K (mg L^−1^ of soil) was determined from a filtered ammonium nitrate extract with flame photometry (MAFF, 1986). Extractable P (mg L^−1^) by the standard Olsen method. Soil pH was measured in a 1:2.5 ratio of H_2_O. Clay content (% < 2 *μ*m) was measured using the pipette method on < 2 mm mineral (peroxide-treated) soil (for further details see (McGrath and Loveland, 1992)).

### 2.4 Spectroscopy

#### 2.4.1 (V)NIR measurements

Whereas spectroscopy measurements for the NSI dataset were taken only on milled samples (as this is the condition of the available stored samples in the NSI), the spectroscopy measurements for the field-scale dataset were taken on in-situ, unprocessed, air-dried and milled samples (Fig. 1). The VNIR spectra from in-situ, unprocessed, and air-dried samples were taken using an ASD FieldSpec 4 spectrometer (Malvern Panalytical Inc., Westborough USA) in the range of 350–2500 nm with a resolution of 3 nm at 700 nm and 10 nm at 1400- and 2100 nm. In-situ measurements were taken with the ASD contact probe after we removed the rubber o-ring and placed a Prolene Thin Film (Chemplex Industries Inc., Florida USA) across the glass sampling interface to avoid contamination. In-situ measurements were taken where the top-soil appeared dry and we placed the ASD contact probe on the soil surface, ensuring good contact, without plant residues or stones to take spectral measurements. In-situ measurements were taken at three different locations within the 0.5 m × 0.5 m quadrat used for soil sampling. The measurements on unprocessed samples were taken on the fresh bulked sample, before air-drying, sieving and milling. These samples were placed within a petri-dish and measured with the ASD contact probe. The fresh, unprocessed, bulked samples did not show aggregation and were rather moldable due to their high volumetric water content (ranging from 20% to 45%), resulting in a relatively smooth surface once the sensor was placed on the sample due to compression. Replicates were taken at three different locations within the petri-dish. The measurements on the air-dried samples were performed on the bulked samples before sieving and milling. The bulked sample varied from aggregates that were approximately 5 cm in width to aggregates reduced to powdery soil, the stone content was negligible. A subsample was (re)poured in triplicate into a glass vial and measured with the ASD Muglight. The milled soil samples were pressed into a small well in replicates of three (6 mm across and approximately 1 mm deep) and placed in a Tensor II spectrometer (Bruker scientific, Ettlingen Germany) in the AfSIS spectral laboratory at Rothamsted Research. Its absorbance spectrum in the range 9997–3999 cm^−1^ (1000–2500 nm), i.e. the near infrared (NIR), was measured with a resolution of 4 cm^−1^ (1 nm). The reflectance, *R* of the ASD FieldSpec4 was transformed to optical density (i.e. absorbance, *A*) as $A=\log_{10}\left(1∕R\right)$ to align with the Tensor II measurements. All triplicate measurements were averaged.

The samples from the NSI database were also measured using the Tensor II instrument, spectroscopy measurements were taken on two aliquots of the sample and were averaged.

#### 2.4.2 MIR measurements

We took in-situ, unprocessed and air-dried MIR measurements using the Agilent 4300 FTIR spectrometer (Agilent Technologies, Santa Clara USA) in the range of 4000–650 cm^−1^ (2500–15 385 nm) with a resolution of 4–16 cm^−1^ (15–62 nm). The in-situ MIR measurements were taken at the same locations as the in-situ VNIR measurements. Equally to the (V)NIR measurements, the MIR measurements for the unprocessed and air-dried samples were taken within a petri-dish and replicate measurements were taken at three different locations. For the milled soil samples, each sub-sample’s mid infrared (MIR) spectrum in the range 4000–600 cm^−1^ (2500–16 666 nm) was recorded on the Tensor II with a resolution of 4 cm^−1^ (16.6 nm). The same well plates with soil aliquots prepared for NIR measurements were used by switching the light source on the Tensor II to MIR. The procedure was repeated for the measurements on the NSI samples.

#### 2.4.3 Spectral pre-processing

All spectra were smoothed to remove noise using the Savitzky–Golay filter (Savitzky, 1964) with a third-order polynomial in a moving window of 11. Subsequently, all spectra have been transformed to their standard normal variate and were subject to 1^*st*^ order derivatization. The derivatives for the (V)NIR spectra were computed with a filter length of 11 (i.e. the spacing between points over which the derivative is computed), a segment size of 31 (i.e. the range over which the points are averaged). Subsequently, two column regions in the (V)NIR spectra were removed as these correspond to moisture absorption bands: (7900–6849 cm^−1^) and (5587–5102 cm^−1^), respectively (Bowers and Hanks, 1965). For the MIR spectra, we used a filter length of 11 and a segment size of 8. The atmospheric CO_2_ bands were removed in the region 2430–2260 cm^−1^ for the MIR spectra (Sandford and Allamandola, 1990).

### 2.5 Data-subsetting and selection of spiking subset by the (V)NIRMIR spectra

The field-scale dataset was split into a dataset for calibration (75%) and validation (25%). We followed the standard procedure where samples are selected to span the range of soil variation anticipated across the samples. This was done using the Kennard-Stone sampling algorithm on the euclidean distance of the 1^*st*^ derivative (V)NIRMIR spectra from milled samples, to select a subset of 75% that represented the field-scale dataset spectrally (Kennard and Stone, 1969).

The spiking methodology comprised two main steps. Firstly, we took 10% of our milled calibration field-scale dataset as a spiking subset (*n* = 7). Again these were chosen by Kennard-Stone sampling. Second, we applied additional weighting to the spiking subset when we regressed the spectra to laboratory reference values. Weighting was applied by adding the spiking subset *m* times, where *m* was the ratio between the size of the spectral library and the spiking subset (Table 1) (Guerrero et al., 2014).

**Table 1:** Summary statistics of the spiking subset and the spectral libraries used to regress laboratory reference values to soil spectra with partial least squares methods.

Dataset	Property	*n*	*m*	Mean	Median	Std dev.	Min	Max	Range	Skew
	Organic C/g kg^−1^			13.39	15.29	5.07	6.26	20.41	14.15	-0.15
Spiking	pH	7	–	7.41	7.51	0.27	6.82	7.63	0.81	-1.32
	Clay/%			33.90	36.66	6.90	22.70	40.04	17.34	-0.63
	P/mg kg^−1^			49.27	42.30	20.38	28.35	86.70	58.35	-1.11
	K/mg kg^−1^			295.82	262.77	190.39	115.41	690.04	574.63	1.08
Regional library	Organic C/g kg^−1^	158	22	5.83	2.80	7.24	0.70	56.40	55.70	3.28
	pH	158	22	7.20	7.50	0.74	4.60	8.20	3.60	-1.34
	Clay/%	127	18	30.76	28.70	14.59	3.00	73.20	70.20	0.36
	P/mg kg^−1^	158	22	37.12	30.50	26.74	4.00	162.00	158.00	1.94
	K/mg kg^−1^	158	22	335.04	305.00	261.13	28.00	2776.00	2748.00	5.23
Stratified library	Organic C/g kg^−1^	109	15	12.19	9.20	10.29	0.70	56.40	55.70	1.59
	pH	108	15	6.53	6.90	1.12	3.60	8.00	4.40	-0.74
	Clay/%	62	8	29.28	26.50	16.26	3.00	73.20	70.20	0.36
	P/mg kg^−1^	108	15	31.91	29.00	20.91	2.00	120.00	118.00	1.33
	K/mg kg^−1^	108	15	249.09	205.00	182.47	21.00	1066.00	1045.00	1.46

### 2.6 Partial least squares regression and model validation

Partial least squares methods were used to regress the absorbance measurements against the wet chemistry reference values. The partial least squares (kernel) algorithm selects orthogonal components that maximize the covariance between the predictor (spectral matrix) and the response (wet chemistry data). We performed a leave-one-out cross validation with the calibration dataset to gain the root mean squared error (RMSE). To avoid over fitting we allowed our models to have a maximum of fifteen components. The number of components retained was equal to the model that gave the lowest RMSE in the cross-validation. For more robust comparison across literature studies, we additionally include the ratio of performance to inter-quartile range (RPIQ). This method provides a standardized metric using the inter-quartile range of the observed data and is recommended by Bellon-Maurel et al. (2010) as suitable for IR spectroscopy predictions on skewed response variables. It is described by:

(1) $$\mathrm{RPIQ}=\frac{q_{y_{i}}\left(3\right)-q_{y_{i}}\left(1\right)}{\sqrt{\frac{\sum {\left(y_{i}-\overset{ˆ}{y_{i}}\right)}^{2}}{N}}}$$

where *y* and $\overset{ˆ}{y}$ are the observed and predicted data for the *i*^*th*^ observation, *N* the total number of samples, $q_{y_{i}}\left(3\right)$ the 3^*rd*^- and $q_{y_{i}}\left(1\right)$ the 1^*st*^-quantile of the observed data. We further computed the prediction bias as the mean of $\left(y-\overset{ˆ}{y}\right)$.

The predictions from different sample conditions and those from spectral libraries were then evaluated on the field-scale validation set. PLSR residuals of the spectral libraries were evaluated for each individual model to assess for the presence of outliers in the spectral library due to subsetting of the NSI by stratification or region. After evaluating the PLSR standardized residuals from the spectral libraries (both with and without spiking) for each soil property, we removed data points that we considered to be outliers. Cut-off values of − 3 and 3 for the standardized residuals were used to remove observations.

### 2.7 Model-averaging of PLSR predictions and their evaluation

Combining predictions from multiple sensors can lead to better accuracy. The PLSR predictions from (V)NIR, MIR matrices for each property were used for an ordinary least squares (OLS) multiple regression, known as the Granger–Ramanathan averaging method (Granger and Ramanathan, 1984). The OLS regression in its general form is

(2) $$Y\;=\;w_{0}+w_{1}z_{1}+\cdots +w_{k}z_{k}\;,$$

where **Y** is a vector of random observed values, **z** is a vector of PLS predictions, the *w*_*i*_, *i* = 1, 2, …, *k*, are weighting coefficients of the *k* individual predictors included in the regression. This equation was solved for the intercept (*w*_0_) and the *k* coefficients for each of the spectral matrix combinations (**z**). The intercepts correct for bias if one of the individual predictors is biased.

To evaluate predictions from in-situ, unprocessed, air-dried and milled samples, we compared models from (V)NIR, MIR and their model average (V)NIRMIR. Since model-averaging gave consistent equal- or improved predictions from milled samples, we evaluated the spectral library predictions on the model averaged (V)NIRMIR predictions only.

Analysis was done using the following R packages: spectral processing using **prospectr** (Stevens and Ramirez-Lopez, 2020) and partial least squares regression using **pls**(Bjørn-Helge et al., 2019). Kennard-Stone sampling using **resemble**(Ramirez-Lopez and Stevens, 2016), Granger–Ramanathan averaging using **GeomComb**(Weiss and Roetzer, 2016), Lin's Concordance correlation coefficienty using **epiR** (Stevenson, 2020). Graphics were created with **ggplot2**(Wickham, 2016) and maps using QGIS3 (QGIS development team, 2019).

## 3. Results and discussion

### 3.1 The selection of representative samples for calibration

Soil spectroscopy is applied under the assumption that the calibration dataset is representative of the target population. It is therefore important that the calibration set spans the range of wet chemistry values in the validation dataset. This was the case for all of the soil properties we considered except for available P and K, which had a slightly lower minimum in the validation data compared to the calibration data (Table 2).

**Table 2:** Summary statistics of the field-scale dataset that was used to regress laboratory reference values to soil spectra with partial least squares methods.

Dataset	Property	*n*	Mean	Median	Std dev.	Min	Max	Range	Skew
	Organic C/g kg^−1^		12.99	12.94	3.44	6.26	20.41	14.15	-0.02
Field-scale	pH	97	7.31	7.48	0.46	5.37	7.77	2.40	-2.29
	Clay/%		35.80	36.57	4.69	22.70	44.63	21.94	-0.38
	P/mg kg^−1^		46.17	44.65	12.28	25.32	86.70	61.38	0.72
	K/mg kg^−1^		283.34	278.93	120.41	86.65	705.22	618.57	0.86
	Organic C/g kg^−1^		13.06	13.15	3.43	6.26	20.41	14.15	-0.04
Calibration	pH	73	7.32	7.49	0.46	5.37	7.74	2.37	-2.41
	Clay/%		35.59	36.19	4.74	22.70	44.63	21.94	-0.46
	P/mg kg^−1^		47.42	45.83	13.15	27.12	86.70	59.58	0.68
	K/mg kg^−1^		289.46	274.93	125.87	91.97	705.22	613.25	0.97
	Organic C/g kg^−1^		12.79	12.45	3.54	6.58	18.08	11.50	0.05
Validation	pH	24	7.27	7.45	0.47	5.68	7.77	2.09	-1.84
	Clay/%		36.46	37.26	4.55	30.14	44.38	14.24	-0.07
	P/mg kg^−1^		42.34	44.22	8.25	25.32	54.20	28.88	-0.77
	K/mg kg^−1^		284.94	298.82	104.40	86.65	513.25	426.60	0.14

Kennard-stone sampling was performed on the combined (V)NIRMIR spectra to select 75% of the samples for calibration and 25% for validation (see method Section 2.5).

The spectral libraries subsetted from the NSI captured the range of wet chemistry data in the field-scale dataset (Tables 1 and 2). However, for all soil properties the distribution differed between the field-scale dataset and the spectral libraries. The spiking subset selected by the Kennard-Stone algorithm encompassed the complete range of the calibration dataset for organic carbon only. A comparable, but incomplete, range was selected for clay, available P and K (Tables 1 and 2). The pH distribution was not well captured in the spiking subset, with a range of 0.81 in the spiking subset compared to the range of 2.37 in the calibration dataset.

### 3.2 The effect of sample processing on soil property prediction accuracy using (V)NIR, MIR and (V)NIRMIR

**Fig. 3:**
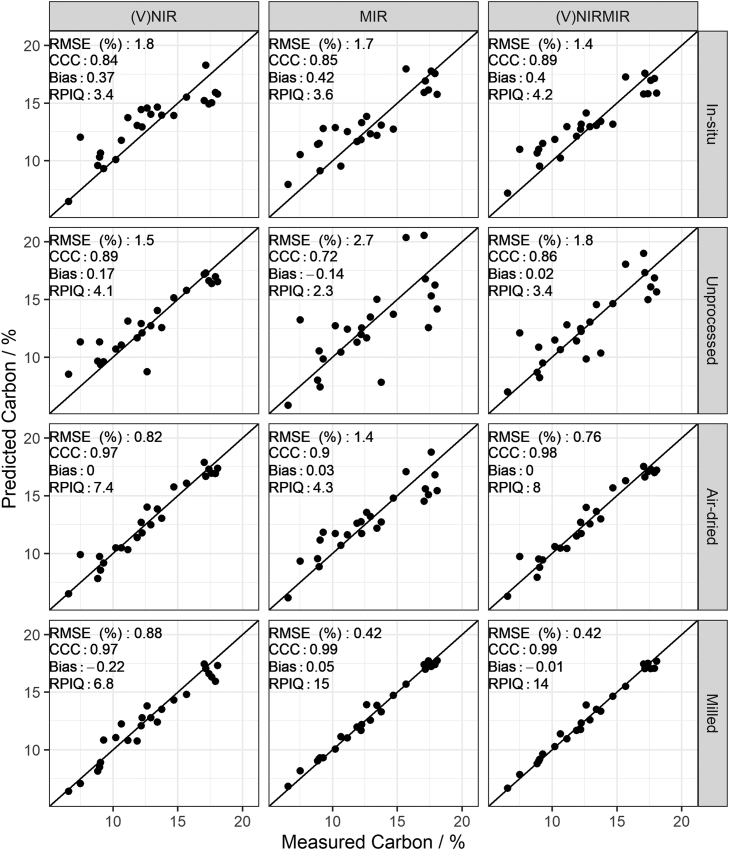
Predicted versus measured organic carbon by partial least squares regression under different soil conditions (in-situ, unprocessed, air-dried and milled) for (V)NIR, MIR, and (V)NIRMIR. RMSE (%): root mean squared error, RPIQ: ratio of performance to inter-quartile range. Prediction models for the top three rows are based on spectra made by handheld spectrometers whereas the models in the bottom row are based on benchtop spectrometer data (details in method Section 2.4).

**Fig. 4:**
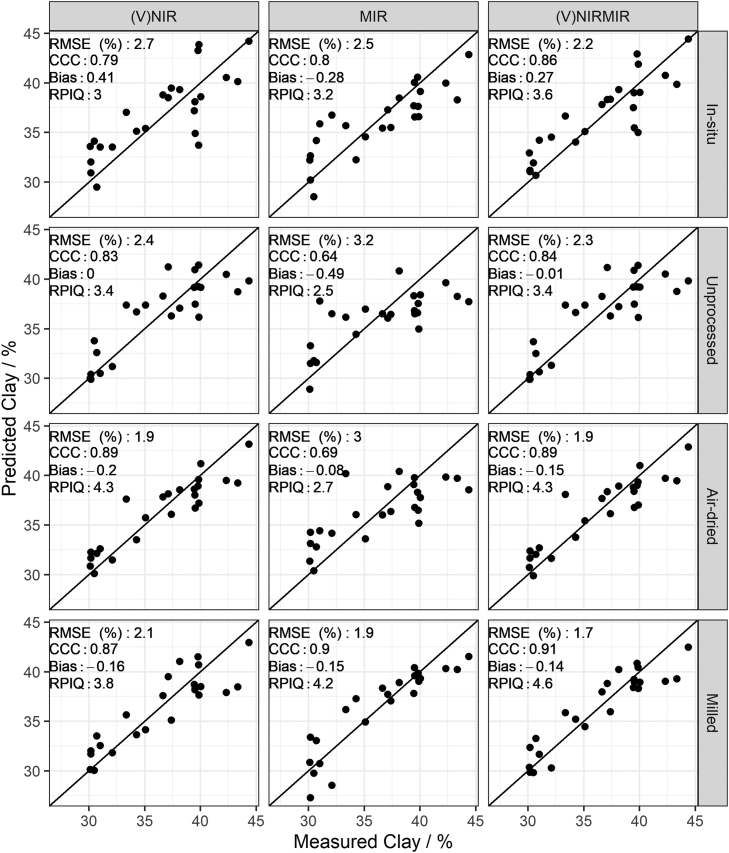
Predicted versus measured clay by partial least squares regression under different soil conditions (in-situ, unprocessed, air-dried and milled) for (V)NIR, MIR, and (V)NIRMIR. RMSE (%): root mean squared error, RPIQ: ratio of performance to inter-quartile range. Prediction models for the top three rows are based on spectra made by handheld spectrometers whereas the models in the bottom row are based on benchtop spectrometer data (details in method Section 2.4).

**Fig. 5:**
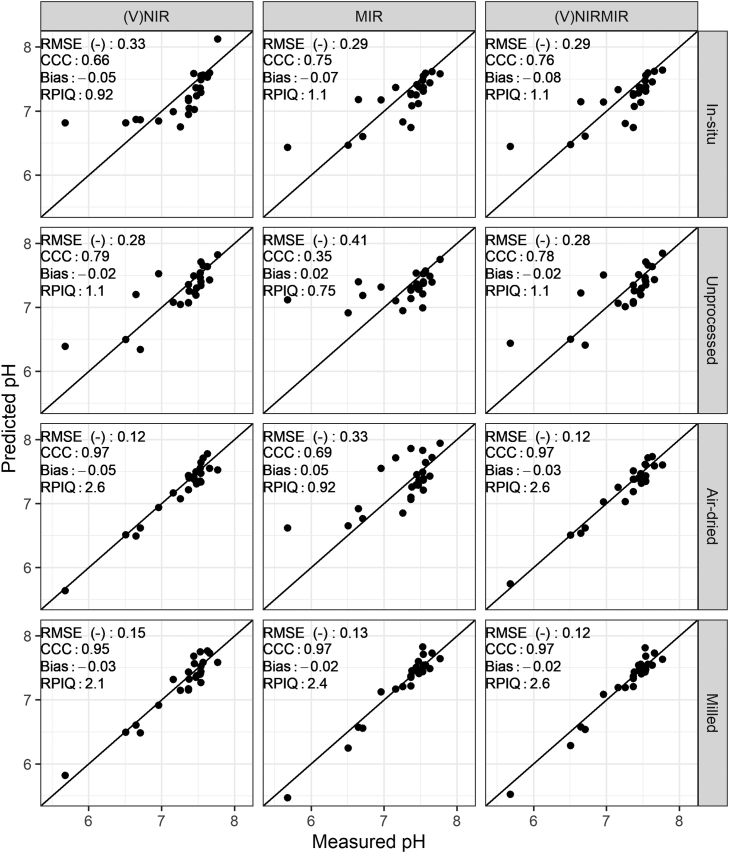
Predicted versus measured pH by partial least squares regression under different soil conditions (in-situ, unprocessed, air-dried and milled) for (V)NIR, MIR, and (V)NIRMIR. RMSE (-): root mean squared error, RPIQ: ratio of performance to inter-quartile range. Prediction models for the top three rows are based on spectra made by handheld spectrometers whereas the models in the bottom row are based on benchtop spectrometer data (details in method Section 2.4).

**Fig. 6:**
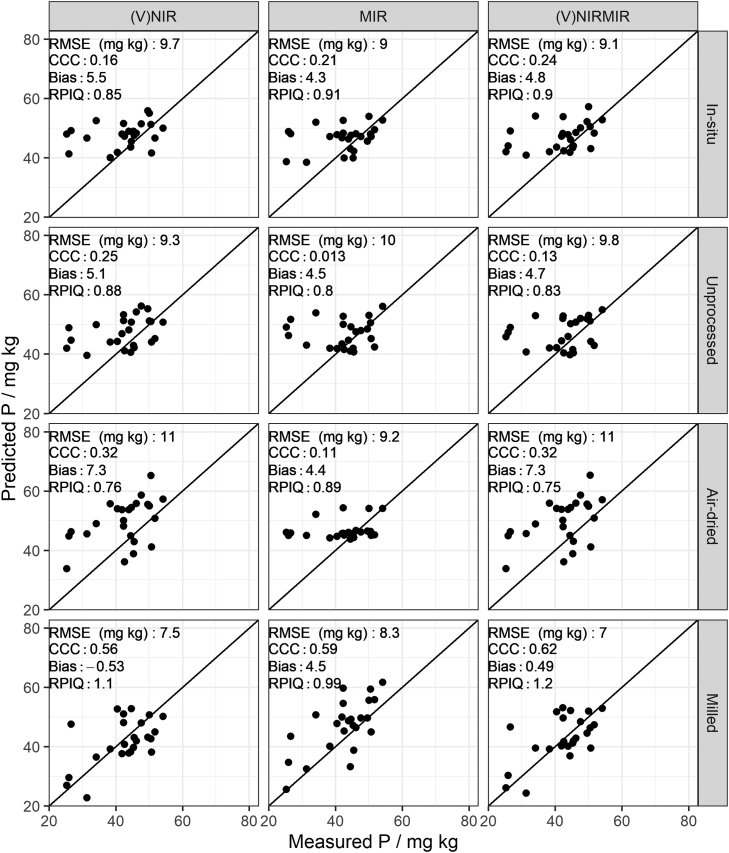
Predicted versus measured available P by partial least squares regression under different soil conditions (in-situ, unprocessed, air-dried and milled) for (V)NIR, MIR, and (V)NIRMIR. RMSE (mg kg): root mean squared error, RPIQ: ratio of performance to inter-quartile range. Prediction models for the top three rows are based on spectra made by handheld spectrometers whereas the models in the bottom row are based on benchtop spectrometer data (details in method Section 2.4).

**Fig. 7:**
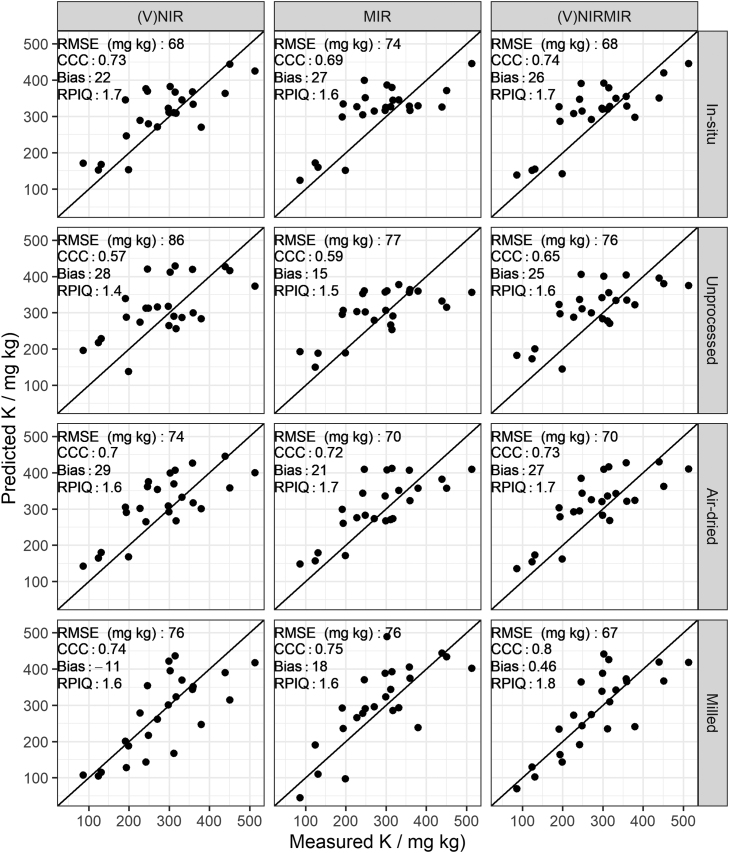
Predicted versus measured available K by partial least squares regression under different soil conditions (in-situ, unprocessed, air-dried and milled) for (V)NIR, MIR, and (V)NIRMIR. RMSE (mg kg): root mean squared error, RPIQ: ratio of performance to inter-quartile range. Prediction models for the top three rows are based on spectra made by handheld spectrometers whereas the models in the bottom row are based on benchtop spectrometer data (details in method Section 2.4).

As expected, the effort of sample processing and homogenisation, i.e. air-drying and milling, led to the best predictions in all soil properties. The RPIQ values for organic carbon, clay and pH predictions from milled samples compare favourably to existing literature (Fig. 3, Fig. 4, Fig. 5). For example, several studies list RPIQ values that range from: 2.49 to 3.6 for organic carbon, 1.55–2.25 for pH and 3.88–6.4 for clay (Nocita et al., 2014, Terra et al., 2015, Clairotte et al., 2016, Hermansen et al., 2016, O’Rourke et al., 2016). We note that most of the studies listed predicted soil properties at a different geographical scale, hence comparison needs to be viewed with caution. Although the RPIQ metric allows for better comparison, variances are dependent on the concentration of the target variable which in turn depends on geographical extent and soil variation present. Available P and K could be moderately predicted only under milled sample conditions (Fig. 6 and Fig. 7).

#### 3.2.1 Contrasting (V)NIR, MIR and (V)NIRMIR predictions

Model-averaged (V)NIRMIR led in most cases to either equal or consistent, albeit small, improvement in predictions compared to (V)NIR and MIR predictions by themselves (Fig. 3, Fig. 6, Fig. 7, Fig. 4, Fig. 5). The limited benefit of (V)NIRMIR compared to MIR for milled samples has been previously observed by Clairotte et al. (2016) in their study on soil organic carbon. Our results indicated that model-averaging did improve accuracy for organic carbon predictions from in-situ and air-dried samples in particular (Fig. 3). Granger-Ramanathan averaging ensured in most cases that the (V)NIRMIR predictions were more accurate or comparable compared to the best predictions of either (V)NIR or MIR, this has also been shown in other studies (O’Rourke et al., 2016). We further note that model-averaging improved prediction by reducing bias, demonstrated in our study for available K predictions from milled samples (Fig. 7).

For in-situ and milled sample conditions, predictions of organic carbon, pH and clay based on MIR measurements outperformed (V)NIR predictions. (V)NIR predictions of organic carbon, pH and clay outperformed those from MIR for unprocessed or air-dried conditions (Fig. 3, Fig. 4, Fig. 5). Over all four sample conditions, predictions from milled samples did not always guarantee the best accuracy. (V)NIR predictions from air-dried samples of pH were equal to the most accurate predictions from milled samples (RPIQ = 2.6 for both). Clay, pH and organic carbon predictions from air-dried samples outperformed those from milled samples for the (V)NIR range only (Fig. 3, Fig. 4, Fig. 5).

Nduwamungu et al. (2009) did not find improvements in predictions from < 2 mm soil samples for the NIR range. Both LeGuillou et al. (2015) and Wijewardane et al. (2020) reported that predictions from fine ground samples always outperformed those from non-fine ground for the MIR region. Results in this study align with the literature. For the (V)NIR range, milling did not strictly show improvement in predictions. However, milling always led to the most accurate predictions from MIR spectra.

#### 3.2.2 Spectrometer differences and sample heterogeneity

Observed differences in prediction accuracy cannot be solely attributed to sample conditions because the spectra from handheld spectrometers are not directly comparable to those measured by benchtop spectrometers.

MIR predictions from in-situ, unprocessed and air-dried samples underperformed compared to MIR predictions from milled samples. This can be explained in part by the small support size of the FTIR 4300 sampling interface (2–3 mm), which results in problems to scan a representative area of the soil sample (Reeves, 2010). Ji et al. (2016) found that small-scale soil heterogeneity and electrical noise contributed up to 50% of the total prediction error of soil properties in their in-situ MIR study. Hutengs et al. (2018) found that the MIR portable spectrometer used in this study measured spectra with the same accuracy as a DRIFT benchtop spectrometer (equivalent to the Tensor II in this study) for milled samples, particularly when replicate measurements with the handheld instrument were taken at different locations to account for the small support size of the sampling interface.

For the (V)NIR spectra, predictions of organic carbon and pH from air-dried samples outperformed those from milled samples. Several studies reported that (V)NIR predictions within the field outperformed those under lab conditions (Gras et al., 2014, Stevens et al., 2008). Stevens et al. (2008) explain their results are due to the dryness of the soil, soil roughness and vegetation cover associated with their in-situ measurements. Spectral processing to mitigate confounding effects is also mentioned as a potential contributing factor to good predictions from in-situ reflectance measurements. Although the benchtop spectrometer used to collect milled (V)NIR spectra has a reduced wavelength range (excluding the VIS region), no large differences between benchtop and laboratory (V)NIR spectrometers have been reported when compared on the same sample conditions (Hodge and Sudduth, 2012, Knadel et al., 2013, Lopo et al., 2016). However, the usefulness of the VIS region, particularly for organic matter predictions, has been pointed out in multiple studies (Islam et al., 2003, Fystro, 2002). Conversely, Chang et al. (2001) and Dunn et al. (2002) reported poor predictions for organic matter in their studies from VIS. The VIS region also relates to texture, structure, moisture and mineralogy. It appears that the soil’s brightness as a predictor for soil properties has an application within limited geological types/parent materials (Stenberg and Viscarra Rossel et al., 2010). One other factor that could contribute to increased prediction accuracy is that (V)NIR measurements from the handheld spectrometer tend to smooth effects of sample heterogeneity on the spectrum, since measurements were averaged over a larger surface area (10 mm spot size for the contact probe and 12 mm for the mug light) (ASD Accessories User Manual, 2021).

Although the predictions from unprocessed samples were the least accurate, the effect of soil moisture content was not as large as we expected based on the range of mass-based percent (20–45%). Soil moisture reduces total reflectance, particularly for the H_2_O bands, where the magnitude of this relation changes between different soil types (Bowers and Hanks, 1965, Minasny et al., 2009). Although this effect generally reduces robustness of a calibration, in our case-study the timing of sampling might have enhanced a distinction between spectra from the two soil types due to their difference in water holding capacity.

### 3.3 The use of spectral libraries compared with field-scale calibration models

**Fig. 8:**
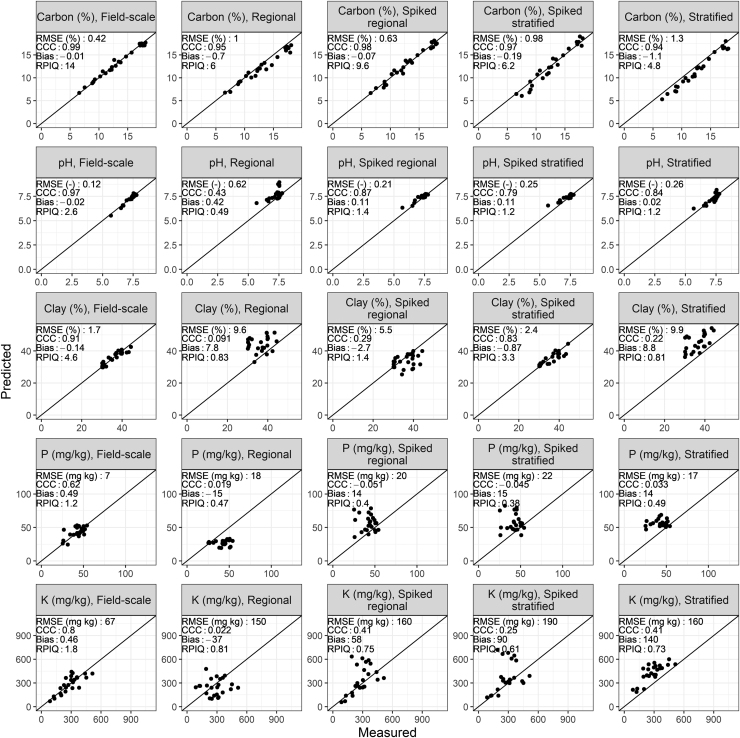
Predicted versus measured organic carbon, pH, clay, available P and K from the spectral libraries and spiked spectral libraries and the field-scale dataset. Models and predictions were performed using milled samples and by model-averaging predictions from (V)NIR and MIR spectra. RMSE: root mean squared error, RPIQ: ratio of performance to inter-quartile range.

Across the variables considered, unsurprisingly the field-scale calibration model led to the best predictions (Fig. 8). Comparing our regional and stratified spectral libraries (without spiking), the regional library performed best for organic carbon whereas the stratified library performed best for pH. Organic carbon predictions from the unspiked regional library showed good precision (i.e. they captured the range) but poorer accuracy, i.e. large RMSE and bias (Fig. 8). Predictions for pH from the unspiked stratified library showed moderate precision and accuracy (Unspiked stratified: RPIQ = 1.2, Bias = 0.02). Clay, available P and K showed poor results for spectral libraries without spiking (Fig. 8).

Our poor clay predictions contrast with the literature, for example, Waiser et al. (2007) state that kaolinite, smectite and muscovite minerals have distinct spectral features which leads to accurate predictions. In our study, the poor clay predictions were likely due to different laboratory methods and the high organic matter content of the soil: samples from the National Soil Inventory were pre-treated with H_2_O_2_ and measured by the pipette method. The field-scale dataset samples were not pre-treated due to their high organic carbon content and were measured by laser diffraction. The confounding effect of different laboratory methods on prediction performance of spectral libraries has been mentioned within the literature previously ( Viscarra Rossel et al., 2016). Differences between laboratory methods also occurred for available P and K, in particular the fact that the NSI extractions were standardised by volume of soil rather than their weight (McGrath and Loveland, 1992). Given the organic nature and therefore low bulk density of the two soil types in the case study area, the standardisation by volume will have affected the comparison to the field-scale dataset (standardised by weight). Additionally, available P and K are known to have weak or no spectral features in the IR region (Kuang et al., 2012).

Spectral library predictions of organic carbon and clay improved substantially once spiked (Fig. 8). Clay predictions were still poor from the spiked regional library compared to the field-scale dataset with a large bias (Spiked regional: Bias = 2.7, Field-scale: Bias = 0.02). Spiking only improved predictions for pH from the regional library (Unspiked regional: RPIQ = 0.49, spiked regional: RPIQ = 1.4). Once spiked, organic carbon and pH predictions from the regional library outperformed the stratified library, suggesting that geographical representation, rather than soil type in the spectral library is more representative of the relation between these properties and spectral reflectance. This potentially reflects a regional soil signature caused by a specific land use or management in the area (the case-study area is used for outdoor horticulture). Clay predictions from the spiked stratified library outperformed those from the spiked regional library. Clay is unaffected by local management and more closely related to soil lithology and parent material, which could explain the better representation of the stratified library for this property.

### 3.4 Contrasting time and cost implications of spectroscopy predictions from spectral libraries and samples with reduced processing

The decision whether to reduce sample processing or use spectral libraries depends on the soil property of interest. In our analysis we found that neither reduction in effort would allow accurate prediction of P or K but both showed promise for predictions of organic carbon, clay and pH. For these variables, the choice of which approach to use in practice will depend on the accuracy required, the number of prediction samples needed and the costs associated with field sampling, preparation and handling and laboratory costs. This will be case study specific, but here we place the relative differences in uncertainty in context of the data acquisition process.

For example, our results showed that RMSEs for organic carbon content from spiked spectral libraries (RMSE (%) = 0.63–0.98) were lower compared to the lowest RMSEs under in-situ (RMSE (%) = 1.4) and unprocessed (RMSE (%) = 1.5) sample conditions. The lowest RMSE for organic carbon predictions from air-dried samples (RMSE (%) = 0.76) lay in between the spiked stratified and regional library predictions. However, the use of spiked spectral libraries still requires sampling a field-scale dataset where samples need to be air-dried, sieved and milled so they are comparative to the samples from the library.

Prediction accuracy under in-situ, unprocessed and air-dried conditions was good but calibration samples had to be analysed with wet chemistry data compared to the spectral library approach, where wet chemistry data was already available. In some situations, the cost of a greater number of samples to be processed and analysed by wet chemistry could be offset by reduced hours spent on field sampling (in-situ) or handling of the samples (unprocessed and air-dried) (Fig. 1). For example, for (V)NIR predictions only, there was no loss in accuracy for organic carbon, pH and clay predictions from air-dried samples. Hence, the benefits of milling became redundant. Similarly there is a trade-off between the two approaches in terms of laboratory, sampling and handling costs occurring for clay predictions. In our study, the best clay predictions under in-situ and unprocessed sample conditions were roughly equal to those from the stratified spiked spectral library (in-situ: RPIQ = 3.6, unprocessed: RPIQ = 3.4, spiked stratified: RPIQ = 3.3). Clay predictions from air-dried conditions approximated those of the milled field-scale dataset (air-dried: RPIQ = 4.3, milled: RPIQ = 4.6).

One should of course consider whether the additional loss in accuracy affects the value of the final dataset created from soil property predictions. For example, predictions with reduced accuracy can be of practical use depending on the available budget and purpose of the analysis. An error of 0.12 units of pH (predictions from air-dried or milled samples) in determining liming requirements for an agricultural field could lead to an erroneous under or over application of 1.5 t limestone per ha^−1^. Whether variable rate liming under this condition is cost-effective compared with a field average will depend on specific circumstances of the subfield variation and the price of limestone. Equally this question can be asked for predictions from different sample conditions or spectral libraries that showed a higher error variance.

## 4 Conclusions

This study contrasted the magnitude of loss in accuracy, relative to field-scale predictions on milled samples, by either reduced sample processing or the use of spectral libraries. The results showed that there is potential to reduce time and cost of using near- and mid-infrared spectra to predict soil organic carbon, clay and pH. We found that reduced sample processing lowered the ratio of performance to inter-quartile range (RPIQ) by 0–76%. The use of spectral libraries reduced RPIQ of predictions by 54–82% and was reduced in the range of 29–70% for predictions when spectral libraries were spiked. The reduction in uncertainty was specific to the combination of soil property and sensor analysed. We conclude that the decision about which approach to use will depend on the case in question because implications of cost and uncertainty will vary from case to case. This study provides insight into the expected differences in prediction accuracy, relative to field-scale predictions from milled samples, from spectra measured under reduced sample processing and the use of spectral libraries. It further discusses which factors need to be taken into consideration to reduce effort for developing field-scale calibrations with near- and mid-infrared soil spectra.

## Declaration of Competing Interest

The authors declare that they have no known competing financial interests or personal relationships that could have appeared to influence the work reported in this paper.

## Data Availability

The data the National Soil Inventory are available from Cranfield University. Restrictions apply to the availability of these data, which were used under license for this study. For enquiries about data availability contact nsridata@cranfield.ac.uk. The field-scale dataset will be made available in the open access repository of Cranfield University under DOI: 10.17862/cranfield.rd.16719823 together with the associated R code to perform the analysis under DOI: 10.17862/cranfield.rd.16732891.
