# Supplementary material for: Comparing the effect of different sample conditions and spectral libraries on the prediction accuracy of soil properties from near- and mid-infrared spectra at the field-scale
Source: Soil Tillage Res. 2022 Jan;215:105196. doi: 10.1016/j.still.2021.105196 (PMC8785126; doi:10.1016/j.still.2021.105196)
Supplement: Supplementary file 1 — Supplementary material [file mmc1.pdf]

Supplementary material

Comparing the effect of different sample conditions and spectral libraries on the prediction accuracy of soil properties from near- and mid-infrared spectra at the field-scale

T.S. Breure[1,2] · **J.M. Prout**[1,2] · **S.M. Haefele**[1] · **A.E. Milne**[1] · **J.A. Hannam**[2] · **S. Moreno-Rojas**[3] · **R. Corstanje**[2]

Corresponding author: T.S. Breure

timo.breure@rothamsted.ac.uk

Telephone: +44 (0)1582 763 133.

[1] Rothamsted Research, Harpenden AL5 2JQ, United Kingdom

[2] Cranfield University, Cranfield, Bedfordshire MK43 0AL, United Kingdom

[3] Gs Growers Ltd, Ely CB7 5TZ, United Kingdom

Notes on the difference in prediction accuracy metrics between the leave-one-out cross-validation (LOOCV) and the independent validation set

Prior to the study we anticipated that the confounding effects of soil moisture content, particle size variation and aggregation would reduce the generalizability of calibration models. Consequently, one would expect a larger discrepancy between accuracy metrics from the LOOCV and those from the independent validation set for predictions from in-situ, unprocessed and air-dried samples. As a reference, we have included the prediction accuracy metrics for the LOOCV calibration and the independent validation set in table format.

Table 1: Prediction accuracy metrics for the leave-one-out cross-validation on the calibration set (Calibration) and the predictions on the independent validation set (Validation). Ncomp: number of components included in the partial least squares regression. RMSE: root mean squared error, CCC: Lin's concordance correlation coefficient, RPIQ: ratio of performance to inter-quartile range.

				Calibration			Validation		
Condition	Property	Sensor	Ncomp	RMSE	RPIQ	Bias	RMSE	RPIQ	Bias
**In-situ**	Organic C/g kg^{-1}	(V)NIR	7	1.80	2.90	0.01	1.80	3.40	0.37
	pH		3	0.35	0.71	0.01	0.33	0.92	−0.05
	Clay/%		8	3.6	1.90	−0.03	2.70	3.00	0.41
	P/mg kg^{-1}		1	15.00	1.20	0.22	9.70	0.85	5.50
	K/mg kg^{-1}		2	120.00	1.10	0.76	68.00	1.70	22.10
**In-situ**	Organic C/g kg^{-1}	MIR	7	1.90	2.70	−0.01	1.70	3.60	0.42
	pH		7	0.33	0.77	0.00	0.29	1.10	−0.07
	Clay/%		5	3.90	1.80	0.05	2.50	3.20	−0.28
	P/mg kg^{-1}		1	17.00	1.10	0.23	9.00	0.91	4.29
	K/mg kg^{-1}		2	140.00	0.94	4.79	74.00	1.60	26.60

Continued from previous page				Calibration			Validation		
Condition	Property	Sensor	Ncomp	RMSE	RPIQ	Bias	RMSE	RPIQ	Bias
**Unprocessed**	Organic C/g kg^{-1}	(V)NIR	8	1.60	3.30	−0.03	1.50	4.10	0.17
	pH		6	0.35	0.71	0.00	0.28	1.10	−0.02
	Clay/%		12	3.20	2.10	0.04	2.40	3.40	0.00
	P/mg kg^{-1}		1	16.00	1.10	−0.50	9.30	0.88	5.06
	K/mg kg^{-1}		4	120.00	1.00	0.21	86.00	1.40	28.1
**Unprocessed**	Organic C/g kg^{-1}	MIR	7	2.20	2.40	0.01	2.70	2.30	−0.14
	pH		3	0.45	0.56	0.00	0.41	0.75	0.02
	Clay/%		2	3.80	1.80	0.07	3.20	2.50	−0.49
	P/mg kg^{-1}		2	20.00	0.89	0.14	10.00	0.80	4.48
	K/mg kg^{-1}		2	160.00	0.81	6.00	77.00	1.50	15.50
**Air-dried**	Organic C/g kg^{-1}	(V)NIR	8	0.98	5.20	0.00	0.82	7.40	0.00
	pH		12	0.21	1.20	0.00	0.12	2.60	−0.05
	Clay/%		8	2.60	2.60	0.04	1.90	4.30	−0.20
	P/mg kg^{-1}		4	12.00	1.50	−0.57	11.00	0.76	7.29
	K/mg kg^{-1}		4	110.00	1.20	−3.06	74.00	1.60	28.70
**Air-dried**	Organic C/g kg^{-1}	MIR	4	2.20	2.30	0.00	1.40	4.30	0.03
	pH		7	0.39	0.63	0.00	0.33	0.92	0.05
	Clay/%		4	4.50	1.50	0.00	3.00	2.70	−0.08
	P/mg kg^{-1}		1	16.00	1.10	0.22	9.20	0.89	4.44
	K/mg kg^{-1}		2	120.00	1.00	2.78	70.00	1.70	21.30

Continued from previous page				Calibration			Validation		
Condition	Property	Sensor	Ncomp	RMSE	RPIQ	Bias	RMSE	RPIQ	Bias
**Milled**	Organic C/g kg^{-1}	(V)NIR	9	1.60	3.10	0.02	0.88	6.80	−0.22
	pH		12	0.27	0.94	0.00	0.15	2.10	−0.03
	Clay/%		11	3.00	2.30	−0.01	2.10	3.80	−0.16
	P/mg kg^{-1}		15	12.00	1.50	−0.10	7.50	1.10	−0.53
	K/mg kg^{-1}		12	110.00	1.20	1.31	76.00	1.60	−11.20
**Milled**	Organic C/g kg^{-1}	MIR	9	1.10	4.60	0.01	0.42	15.00	0.05
	pH		15	0.20	1.30	0.00	0.13	2.40	−0.02
	Clay/%		6	2.10	3.30	0.04	1.90	4.20	−0.15
	P/mg kg^{-1}		13	10.00	1.70	−0.14	8.30	0.99	4.50
	K/mg kg^{-1}		9	98.00	1.30	−1.38	76.00	1.60	18.40
